# The path to find an HIV vaccine

**DOI:** 10.1002/jia2.25749

**Published:** 2021-05-18

**Authors:** Glenda E Gray, Lawrence Corey

**Affiliations:** ^1^ South African Medical Research Council Cape Town South Africa; ^2^ Vaccine and Infectious Disease Division Fred Hutchinson Cancer Research Center Seattle USA

**Keywords:** HIV vaccine, clinical trials, broadly neutralizing antibodies

Since the discovery that a retrovirus, human immunodeficiency virus type one (HIV‐1), causes the acquired immune deficiency syndrome (AIDS), more than 77 million people have become infected with HIV and 37 million have died over the last 40 years [1]. Therapeutic breakthroughs in the form of antiretroviral drugs have ensured the increased survival of adults and children living with HIV, have led to major in‐roads for preventing paediatric HIV infection and produced strategies to deploy antiretrovirals to prevent sexual transmission. Despite these scientific advancements, the HIV pandemic is not under control and continues to devastate the lives of millions of people around the world. The lack of an HIV vaccine underscores the complexity of the immune evasion strategies utilized by HIV. Underlying challenges that scientists face in their endeavour to find a vaccine include the lack of a model for natural immunity, a virus that mutates rapidly, and the inability of the human species to self‐cure HIV and hence define a correlate of protection against naturally occurring HIV infection. HIV vaccine development has become a process of stepwise learning of how to make a vaccine that induces an immune response that is markedly better than the human immune response to HIV. Insights into these processes have occurred when efficacy trials of candidate vaccines have taken place. Scientific advancements, innovation, political will, coupled with the substantial financial investment will continue to be critical in our path to find a vaccine against this virus that is among the biggest killers in history.

To date only one HIV vaccine trial has demonstrated modest efficacy. The RV144 study conducted in Thailand investigated a heterologous prime/boost vaccine regimen consisting of ALVAC HIV (vcp1572) plus AIDSVAX B/E gp120 [2]. With a demonstrated vaccine efficacy of 31.2%, the mechanism of protection was thought to be via non‐neutralizing immune responses from several functional antibody assays such as antibody‐dependent cellular phagocytosis (ADCP) and antibody‐dependent cellular cytotoxicity (ADCC) [3]. In addition, IgG antibodies that recognize the V2 loop in the HIV envelope gp120 appeared to correlate with a reduced acquisition. These findings stood in contrast to traditional vaccine development, which relies on eliciting high levels of broadly neutralizing antibodies as the major mechanism of protection most recently exemplified by the high rate of success of COVID‐19 vaccines [4, 5, 6, 7]. Vaccines against SARS‐CoV‐2 elicit high levels of neutralizing antibody and high‐grade efficacy with a reasonable correlation between the level of neutralizing antibodies and increasing efficacy.

In 2014, the HIV Vaccine Trials Network (HVTN) outlined a strategy to execute a series of large‐scale efficacy trials that would define the approach needed for a successful HIV vaccine. This strategy was broken into two frameworks: *the first* to determine whether broadly protective antibodies could reduce HIV acquisition, that is could a vaccine regimen that elicited high levels of non‐neutralizing antibodies be developed that would enhance the protective efficacy beyond that seen in RV144; *and second*, to discern whether neutralizing antibodies could actually prevent acquisition. As no vaccine as yet has been able to elicit anything but strain‐specific neutralization, the neutralizing antibody approach was to use the passive infusion of broadly neutralizing antibodies brought on by the revolution of B‐cell engineering and cloning to evaluate whether high levels of monoclonal antibodies could reduce HIV acquisition.

Between 2015 and 2019, the HVTN initiated five large‐scale efficacy trials: three in sub‐Saharan Africa and two in North and South America. Three were directed at stimulating non‐neutralizing antibodies and one set of integrated trials evaluated the passive infusion of the monoclonal antibody VRC01 in the antibody‐mediated prevention (AMP) trials. The past 12 months have started to bring in the results of these trials. The earliest returns from non‐neutralizing antibodies have been disappointing. HVTN 702, which was built upon the regimen of RV144, was stopped in January 2020 for lack of efficacy. HVTN 702 was based upon the same regimen used in RV144 except adapted to the subtype C region. Despite evidence of high levels of binding antibodies, ADCP and ADCC activity, no efficacy was noted [8]. The one deficiency in the HVTN 702 trial compared to RV144 was that the regimen induced fewer V2 loop antibodies that RV144. Importantly, two of the other non‐neutralizing trials, HVTN 705 (known as “Imbokodo”) and HVTN 706 (known as “Mosaico”), are currently in progress. The Imbokodo trial, conducted in sub‐Saharan Africa in heterosexual women, tests a diverse set of four synthetically designed envelope proteins in an Ad26 platform aimed to give an increased breadth of immune response in combination with a subtype C gp140 [9] and is due to be analysed for efficacy in July 2021. Its companion trial, Mosaico, enrolling MSM and transgender persons in South America, Mexico and the United States is halfway enrolled. Importantly, the vaccine‐induced immune responses differ considerably from those elicited in HVTN 702, and are non‐neutralizing with different functional levels of response both in T‐cell and humoral immunity. The results of the Imbokodo and Mosaico studies will be critical in the quest to understand whether non‐neutralizing antibodies are capable of inducing protection against HIV.

While the desire to develop neutralizing antibody vaccines to HIV has been there since inception, to date, only strain‐specific immune responses have been elicited by any candidate vaccine. Essentially the inability to elicit broadly neutralizing antibodies to HIV to cover its strain diversity has been a major flaw in the HIV vaccine field for the first 30 years of vaccine development. The quest to overcome this was provided by B‐cell cloning technology, which demonstrated that broadly neutralizing antibodies or antibodies that could neutralize a wide diversity of strains could be isolated from about 15% of HIV‐infected people who had longstanding and often uncontrolled infection [10]. This resulted in the discovery and development of several broadly neutralizing monoclonal antibodies against HIV, many of which operate at distinct areas on the HIV surface and hence could be brought together in cocktails to make highly efficient antiviral combinations similar to what has been achieved with antiretroviral therapy.

To evaluate this concept, the AMP trials, one in southern Africa (HVTN 703/HPTN 081) and the other in the Americas (HVTN 704/HPTN 085), were conducted to determine whether the infusion of broadly neutralizing antibodies targeting the CD4‐binding site called VRC01 could be effective in reducing HIV acquisition. The results of these studies recently published propose the answer was yes, with marked efficacy [11]. However, the virus’s formidable nature was also evident in that it required high levels of neutralization to be effective. Only those viruses extremely sensitive in the in vitro assays were susceptible indicating that large doses of combination monoclonal antibodies to cover the broad spectrum of HIV isolates would be required to advance this concept further.

Most relevant to HIV vaccine development have been recent breakthroughs in developing approaches to initiate the early germline that are the precursors of broadly neutralizing antibodies to the CD4 binding site, MPER, and V3 regions of the virus [12, 13, 14, 15, 16]. All 3 of these regions are important sites for antiviral broadly neutralizing antibodies. These achievements have been made using synthetic nanoparticles to illicit what are called germline antibodies in high frequency, providing optimism that one could then use a boost with other more traditional immune agents to develop a broadly neutralizing vaccine regimen. The addition of mRNA technology to allow faster iteration of candidate immunogens should also provide the field with greater ability to iterate candidate regimens.

The year 2021 brings optimism that the technologies to develop broadly neutralizing vaccines have been developed and major progress in this arena will be achieved. If we can do this, we can apply the momentum, enthusiasm and resources that COVID‐19 vaccines have brought to the world to make the possibility of achieving an HIV vaccine a reality.

HIV Vaccine Awareness Day remains a critical reminder that without this critical biomedical tool, an AIDS‐free generation is unlikely to be achieved. On this day, we also acknowledge and appreciate the many people and their communities who have walked these miles with scientists in the quest to find an effective HIV vaccine.

## Competing interests

The authors have declared no conflict of interest.

## Authors’ contributions

All authors have contributed to the preparation of the manuscript, read and approved the final draft.
